# Release Behavior and Antibacterial Activity of Chitosan/Alginate Blends with *Aloe vera* and Silver Nanoparticles

**DOI:** 10.3390/md15100328

**Published:** 2017-10-24

**Authors:** Luisa Fernanda Gómez Chabala, Claudia Elena Echeverri Cuartas, Martha Elena Londoño López

**Affiliations:** 1 Universidad CES-Grupo de Investigación en Ciencias Farmacéuticas (ICIF-CES), Programa de Química Farmacéutica, Facultad de Ciencias y Biotecnología, Universidad CES, 050021 Medellin, Antioquia, Colombia; 2 Universidad EIA-Grupo de Investigación en Ingeniería Biomédica EIA-CES (GIBEC), Departamento de Ingeniería Biomédica, Las Palmas Campus, Universidad EIA and Universidad CES, 055420 Envigado, Antioquia, Colombia; claudia.echeverri@eia.edu.co (C.E.E.C.); marta.londono@eia.edu.co (M.E.L.L.)

**Keywords:** alginate, chitosan, *Aloe vera*, wound dressing

## Abstract

*Aloe vera* is a perennial plant employed for medical, pharmaceutical and cosmetic purposes that is rich in amino acids, enzymes, vitamins and polysaccharides, which are responsible for its therapeutic properties. Incorporating these properties into a biopolymer film obtained from alginate and chitosan allowed the development of a novel wound dressing with antibacterial capacity and healing effects to integrate the antibacterial capacity of silver nanoparticles with the healing and emollient properties of *Aloe vera* gel. Three alginate-chitosan matrices were obtained through blending methods using different proportions of alginate, chitosan, the *Aloe vera* (AV) gel and silver nanoparticles (AgNps), which were incorporated into the polymeric system through immersion methods. Physical, chemical and antibacterial characteristics were evaluated in each matrix. Interaction between alginate and chitosan was identified using the Fourier transform infrared spectroscopy technique (FTIR), porosity was studied using scanning electron microscopy (SEM), swelling degree was calculated by difference in weight, *Aloe vera* gel release capacity was estimated by applying a drug model (Peppas) and finally antibacterial capacity was evaluated against *S. Aureus* and *P. aeruginosa*. Results show that alginate-chitosan (A (1:3 Chit 1/Alg 1); B (1:3 Chit 1.5/Alg 1) and C (3:1 Chit 1/Alg 1/B12)) matrices with *Aloe vera* (AV) gel and silver nanoparticles (AgNps) described here displayed antibacterial properties and absorption and *Aloe vera* release capacity making it a potential wound dressing for minor injuries.

## 1. Introduction

Traditional treatment for wound healing (dermatological injuries) has been based on gauze or fabric wound dressing. These kinds of procedures have not been a good option because they do not provide optimum humidity conditions, pH and gas exchange to wounds [1]. On the contrary, these conditions could be an optimum medium for bacteria growth, promoting an infection and making the wound healing process slower [2]. To reduce the risk of infection, numerous products with antimicrobial drugs have been developed, such as silver sulfadiazine, nitrofurazone, and others. Silver sulfadiazine cream tends to be unhelpful because when the wound is in direct contact with silver ions, it may cause allergies, skin pigmentation, toxicity and delays in the process of re-epithelialization of tissue [2]. Due to the above, active products obtained from medicinal plants have been emerging as alternatives to reduce these disadvantages, given their therapeutic properties in healing. One of the most representative examples of this is *Aloe vera* [3]. 

*Aloe vera* (*Aloe barbadensis* Miller) is a native plant of the tropics that belongs to the Liliaceae family. It grows in arid soil and is resistant to high temperatures. For years, *Aloe vera* gel has been known for its medicinal properties, given its great source of polysaccharides, such as acemannan, mannan, galactan, glucuronic acid, etc. These mixtures of polysaccharides give *Aloe vera* gel the anti-inflammatory, anti-tumor, immodulatory and antibacterial properties that are responsible for its health properties [4].

In recent years, biopolymer dressings have been developed in order to facilitate use and supply of *Aloe vera* gel in injured areas, being alginate and chitosan among the most representative polymers used, due to their properties. Chitosan is a biodegradable and non-toxic polysaccharide, extracted from shellfish, such as shrimp, crab, *Ganoderma lucidum* and lobster. Moreover, the presence of the amino group in its chemical structure gives the chitosan its antibacterial properties [3,4,5]. For this reason, these have been used in the creation of polymeric systems for medical applications. Ye Ma et al. elaborated a transparent and flexible chitosan crosslinked with glycerin for film encapsulating an antibacterial drug by means of a casting/solvent evaporation method. The matrix showed an improvement in its swelling degree, water vapor permeability and wettability with an increase of glycerol content and presented antibacterial activity against *E. coli* and *S. aureus* [6]. 

Like chitosan, alginate is a natural linear unbranched polysaccharide based on 1,4-linked copolymer of β-d-mannuronic acid and alpha l-guluronic acid residues [7,8], which can be extracted from brown algae and some bacteria such as *P. aeruginosa*. This polymer has been widely used in wound treatments, due to its biocompatibility, biodegradability, hydrophilicity, non-toxicity, non-immunogenicity and its property to form gels and films [8,9]. Likewise, alginate dressings aid in hemostasis as part of the wound healing process and they can trigger human macrophages to produce tumor necrosis factor-α (TNF-α) which initiates inflammatory signals [10]. These alginate characteristics have allowed it to be used as a possible material for medical application especially in the development of wound dressing with therapeutic drugs. Dr. Rezvanian and his colleague formulated a wound dressing of alginate loaded simvastatin to promote would healing obtain through the solvent casting method [7]. Also, the alginate has been used to encapsulate extract obtained from plants. Dr. Pereira et al. created a film composed of alginate and *Aloe vera* gel through solvent-casting method [10].

Due to their properties, these polymers have been applied in developing new wound dressings with antibacterial properties and drug release capacity. Chitosan, honey and gelatin wound dressing have already been applied for their antibacterial capacity against *Staphylococcus aureus* and *Escherichia coli* [11]. Alginate bilayer wound dressings have also been created to encapsulate ibuprofen and allow its slow release. Wound dressings made from natural plant extracts, such as chitosan and *Aloe vera* crosslinked with genipin, have also been explored [3,4].

Electrostatic interaction between chitosan protonated amine groups and alginate carboxylate groups can form a polyelectrolyte complexes (PEC) [12]. Chitosan-alginate PECs maintain the properties of each polymer and show changes in the swelling tendency, furthermore possess structural strength and mechanical stability, which make them suitable for several biomedical applications, such as wound dressings and tissue engineering scaffolds. For this reason, some researchers have dedicated their studies to the development of wound dressing based on PEC. Dr. Kim J.H et al. made a chitosan and sodium alginate sponge (PEC) impregnated with silver sulfadiazine. Essays of equilibrium water and release control of silver sulfadiazine were carried out, finding that reaction number between alginate and chitosan may influence swelling properties and release capacity. Also, this polymeric system presented antibacterial activity against *Pseudomonas aeruginosa* and *Staphylococcus aureus* with a cellular damage reduction compared to silver sulfadiazine [13] Moreover, this crosslinked polymeric system PEC has been developed by Dr. Wanh L and colleagues creating a alginate-chitosan wound dressing (PEC) obtained by aqueous suspensions of chitosan and alginate with CaCl_2_ as the crosslinker. The result was a thin, transparent and flexible matrix, with a low cytotoxic capacity for supporting cell proliferation. Also, the uptake capacity exudated from the matrix showed is related to the pH of the medium, making it a good wound dressing material [14]. Likewise, Dr. Hakan et al. developed and characterized an alginate-chitosan and cerium ion matrix integrating the antimicrobial properties of cerium ions and chitosan. The matrix was obtained by an alginate film crosslinked with cerium (III) solution and chitosan. The result was a wound dressing with antibacterial activity against *Eschericha* coli and *Staphylococcus aureus* [15]. Other researchers have explored different techniques for obtaining a polymeric system PEC, such as Dr. Consantti et al., who prepared a polyelectrolyte complex of alginate and chitosan applying three matrix obtaining techniques: hot air drying, lyophilization and supercritical CO_2_. The best technique among the three was hot drying, which allows it to produce different structures with different porosity scales which improved the swelling degree and drug release [16].

Silver nanoparticles have also been widely used in the medical field due to their biological, chemical, physical properties and antibacterial capacity, which are enhanced at the nanoscale [17]. Sharma et al. demonstrated excellent antibacterial activity against both Gram-negative and Gram-positive bacteria, with more activity against Gram-positive bacteria of an alginate-chitosan blend and silver nanoparticles. Likewise, Montaser and coworkers have developed a wound dressing composite from alginate, silver nanoparticles and nicotinamide with antibacterial properties against *Eschericha coli* and *Staphylococcus aureus* for diabetic burn applications [18].

In this paper, we propose to develop a synergistic association with *Aloe vera*, chitosan, alginate and silver nanoparticles, in order to obtain an active wound dressing with antimicrobial activity and controlled delivery of both *Aloe vera* and AgNps.

## 2. Results and Discussion

### 2.1. ATR-FTIR Spectroscopy 

**Figure 1:**
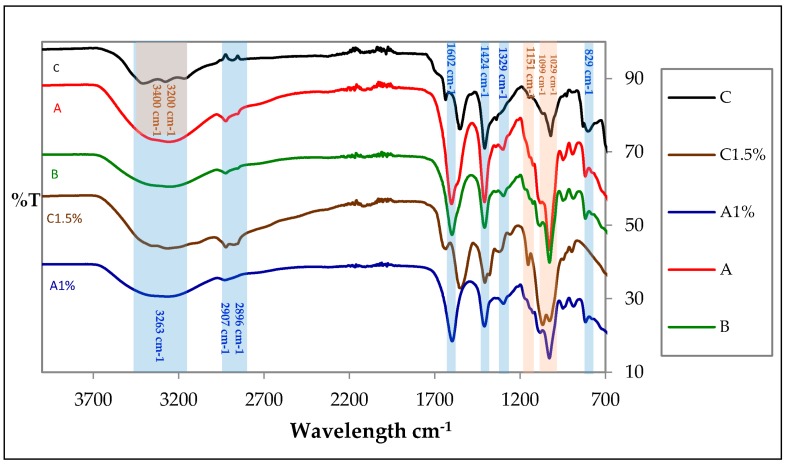
Spectra of A, B and C treatments and blanks Chit 1.5% (Chitosan) and Alg 1% (Alginate).

The attenuated total reflection Fourier transform infrared (ATR-FTIR) technique was used to identify functional groups present and the formation of possible interactions between selected alginate and chitosan treatments: A (1:3 Chit 1/Alg 1) B (1:3 Chit 1.5/Alg 1) and C (3:1 Chit 1/Alg 1/B12) Figure 1 shows spectra of matrices A, B and C; these matrices presented characteristic bands of alginate, such as: two bands of symmetric and asymmetric stretching ion carboxylic (COO–) at 1602 cm^−1^ and 1424 cm^−1^ [19], a C–O stretching at 1319 cm^−1^, a peak of sodium alginate (Na–O) at 821 cm^−1^ [12] and two signals of antisymmetric stretching of glycoside bonds at 1099 cm^−1^ and 1029 cm^−1^, which are also characteristic of chitosan [9,10,11,12,13,14,15,16,17,18,19,20,21].

Similarly, the absorption bands of OH were identified at 3263 cm^−1^, as well as signals of C–H between 2896 cm^−1^ and 2907 cm^−1^ [22]. Moreover, the possible interaction between the negatively charged alginate carbonyl group and the positively charged chitosan amino group [23] might be associated with a small shoulder at 1576 cm^−1^ and the absence of amino group peak at 1560 cm^−1^.

Furthermore, matrix C presented some bands of chitosan, such as: a peak of C–O stretching at 1031 cm^−1^, a weak band of C–N at 1151 cm^−1^ [19,20], a signal of amino group (NH_3_^+^) at 1564 cm^−1^, a signal of amide I at 1644 cm^−1^ [20] and three bands of primary amine stretching between 3400 cm^−1^ and 3200 cm^−1^ [24]. Also, a signal of alginate (Na–O) at 818 cm^−1^. As in the previous case, the absence of some bands in the spectrums of blended polymers (alginate-chitosan) are associated with possible electrostatic interaction between mixed polymers.

### 2.2. SEM Analysis

**Figure 2:**
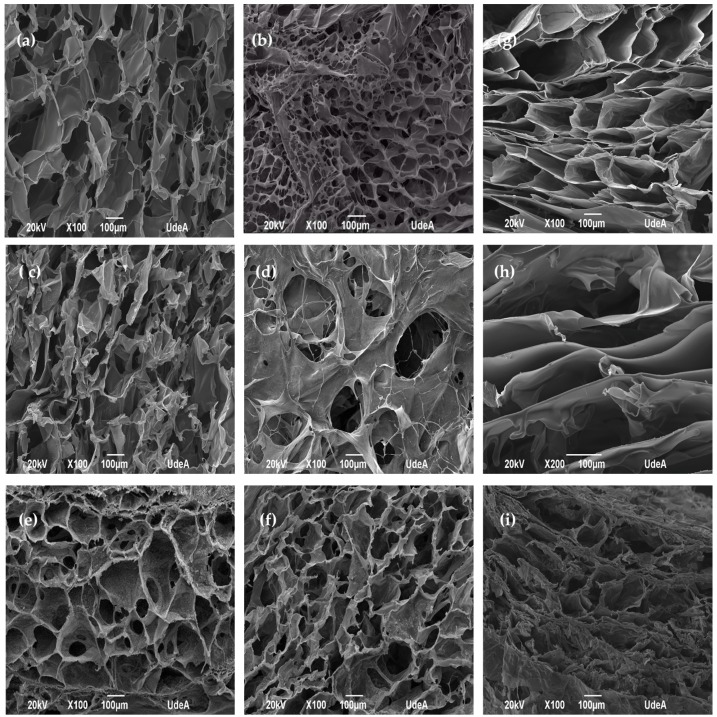
Surface of control alginate-chitosan matrices (**a**) A (1:3 Chit 1/Alg 1), (**c**) B (1:3 Chit 1.5/Alg 1), (**e**) C (3:1 Chit 1/Alg 1/B12) and surface of matrices with *Aloe vera*-silver nanoparticles (AV-AgNps), (**b**) A-AV-AgNps (**d**) B-AV-AgNps, (**f**) C-AV-AgNps. Cross section of alginate-chitosan matrices (**g**) A (1:3 Chit 1/Alg 1), (**h**) B (1:3 Chit 1.5/Alg 1), (**i**) C (3:1 Chit 1/Alg 1/B12.

Figure 2 shows the surface and cross section of control matrices (Figure 2a,c,e) and matrices with *Aloe vera* gel and silver nanoparticles (Figure 2b,d,f). Both matrices displayed high distribution of pores and high porosity as a result of the freeze drying process [15] and pore size between 50 and 200 μm. For this reason, these films become good candidates for cell attachment, especially fibroblasts and a variety of pores on the surface help transport nutrients and oxygen [25,26,27,28]. The freeze drying technique allows us to obtain porous matrices because the water in the system acts as a porogenic agent during the process [29]. The micrographs also show open pores that allow to visualize pores in its interior which indicates the interconnectivity that these pores have in the matrix. Furthermore, the incorporation of *Aloe vera* gel and silver nanoparticles in the polymeric systems alter pore size, as shown in (Figure 2b,d,f). On the other hand, matrix C did not experience alterations when incorporating *Aloe vera* gel and silver nanoparticles [16,30]. However, when comparing the cross-sections of the three treatments (Figure 2g,h,i), the matrices with higher alginate proportions (A and B) presented wider and layer-arranged pores. In the case of matrix C, where the chitosan predominated in the mixture, it displayed narrower pores, producing a more compact structure.

**Figure 3:**
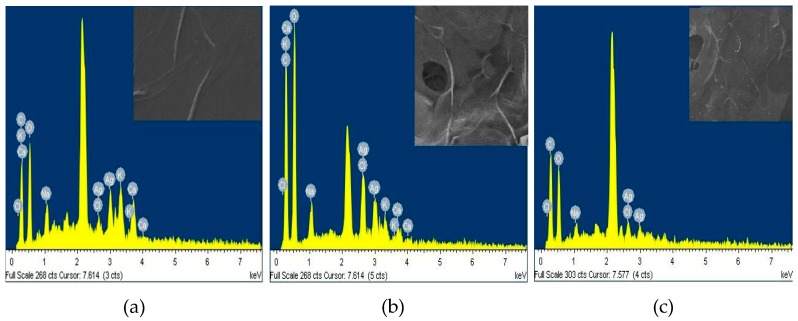
EDS of matrix A loaded with *Aloe vera* gel (AV) and silver nanoparticles (AgNps) (**a**), matrix B loaded with *Aloe vera* gel (AV) and silver nanoparticles (AgNps) (**b**) and matrix C loaded with *Aloe vera* gel (AV) and silver nanoparticles ( AgNps) (**c**).

To detect the silver nanoparticles (AgNpS) in the outer surfaces of matrices, an Energy-dispersive X-ray spectroscopy (EDS) as shown in Figure 3 was carried out. The results reported the weight percentage of silver ions present on the surface of each matrix, finding the following values 8.69% A-AV-AgNps, 4.22% B-AV-AgNps and 4.01% C-AV-AgNps, which evidence the presence of silver nanoparticles in the matrices [27].

### 2.3. Swelling Degree

**Figure 4:**
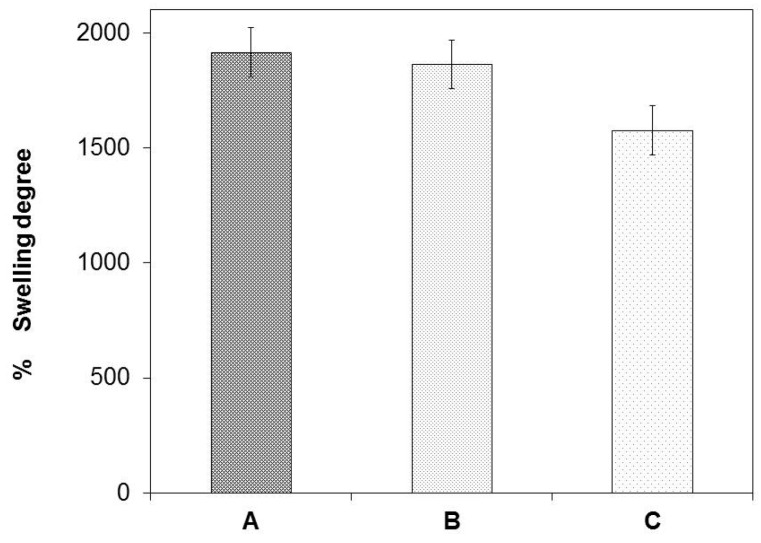
Swelling degrees of matrices A, B and C.

Matrices A and B showed a high swelling degree compared to matrix C, as presented in Figure 4. The swelling degree values of matrices with high alginate ratios were between 1916% and 1864% and the matrix with high chitosan proportion was 1576%.

The capacity developed by the matrices to absorb *Aloe vera* gel is related to high ratios of alginate in the polymeric solution. Matrices A and B displayed wider pores. This characteristic may improve the swelling capacity of the *Aloe vera* gel. On the other hand, matrix C exhibited low swelling capacity. Narrower porous make a more compact structure and reduce its absorption capacity [31].

Berger et.al and Sankalia et al. have reported that ionically crosslinked hydrogels are sensitive to swelling under acidic conditions, and *Aloe vera* gel offers an acidic medium which allows the dissociation of the chitosan and alginate complex. In an acidic medium, the polyacid is neutralized (carboxylate ion –COO^−^ of alginate), leaving the ammonium of chitosan (NH_3_^+^) free, and therefore positive charges appear in the system and produce mutual repulsions and together with the entry of water cause the swelling of matrix [32].

The high chitosan proportion matrix showed a low capacity to absorb *Aloe vera* gel. This behavior could be related to the crosslinking degree of chitosan. As there is an excess of chitosan in the polymer solution, there is an increase in the ammonium group which may interact with more carboxyl groups of the alginate, generating a greater ionic interaction and resulting in a higher density degree in the membrane decreasing its absorption capacity [32].

### 2.4. Release Assay

**Figure 5:**
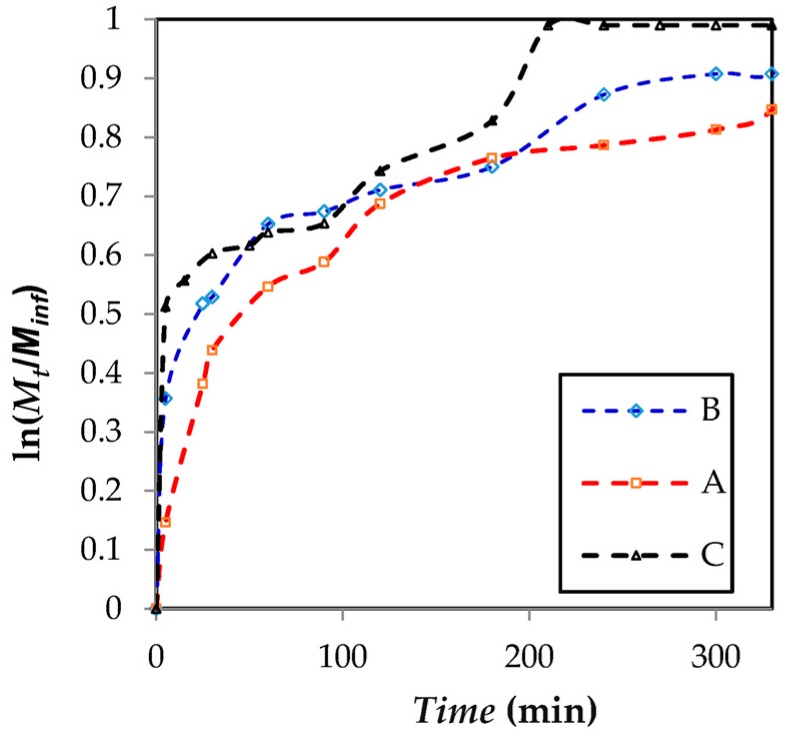
Release profile of *Aloe vera* from a lyophilized matrix of alginate-chitosan.

Figure 5 shows the release of *Aloe vera* gel from each matrix was evaluated in distilled water for a period of 400 min. According to the results, matrices B and C presented a burst effect releasing 37% and 50% of the stored capacity, respectively. Thirty minutes later, matrices B and C released 52% and 50% of *Aloe vera* gel. Matrix A displayed a decrease in the burst effect during the first minutes, releasing about 14% of *Aloe vera* gel and two hours later a release of about 60%. These results show that the flux of *Aloe vera* gel delivered from matrix A was slower than matrices B and C. This behavior could be related to the alginate excess in the polymeric solution. An increase in the proportion of alginate represents a greater number of carboxylate ion available (–COO^−^) which could be protoned during the incorporation of *Aloe vera* gel in the polymeric system. The acid pH of *Aloe vera* gel could neutralize the carboxylate group, resulting in an insoluble gel in water producing a reduction in kinetic release [19,33,34]. However, the matrix with the highest chitosan ratio (3:1 Chi/Alg) increased the number of amine groups protonated in the polymeric system. Figure 5 illustrates a quick release of *Aloe vera* gel in the first few minutes. This behavior is probably due to the amount of amino ions present in the polymer mixture and their solubility in an acidic medium [34].

To understand the release mechanism from the developed matrices, release data was analyzed following the Peppas model. Applying the model to the matrix A data, it found that the release value of *Aloe vera* gel has a good fit to Peppas model, giving an equation ln(*M_t_/M_inf_*) = 0.44 × ln(*t*) − 2.41 with a *R*^2^ = 0.9410 and a release exponent *n* = 0.44 (*p* < 0.05). The release exponent value is close to the theoretical diffusion exponent (*n* = 0.5) (*p* < 0.05) this result indicates that the release mechanism could follow a Fickian diffusion [35,36,37,38]. Similarly, the Peppas model was applied to matrices B and C. It found that the *Aloe vera* gel release from matrix C has a good fit to Peppas model with an equation ln(*M_t_*/*M_inf_*) = 0.26 × ln(*t*) − 1.55 with a *R*^2^ = 0.9316 and a release exponent *n* = 0.26 (*p* < 0.05). In the other case, the *Aloe vera* gel release data from matrix B showed an equation ln(*M_t_/M_inf_*) = 0.10 × ln(*t*) − 1.08 with a *R*^2^ = 0.7691 and a release exponent *n* = 0.10 (*p* < 0.05), demonstrating that the data is far from the applied model.

### 2.5. Antibacterial Activity

In order to determine the antibacterial activity of silver-nanoparticle loaded matrices with *Aloe vera* and *Aloe vera* -AgNps against *S. aureus* (Gram-positive) and *P. aeruginosa* (Gram-negative), the Kirby-Bauer test was conducted using the disk diffusion method. Each of these results were compared with five selected controls: blank matrices (without recharging matrix), *Aloe vera* gel, silver nanoparticles, tetracycline, and gentamicin.

The blank A, B and C matrices did not show inhibition zones, which indicated that the uncharged matrix did not have bactericidal activity against *S. aureus* and *P. aeruginosa.* However, an inhibition zone was identified when the *Aloe vera* gel, AgNps and *Aloe vera* gel-AgNps were incorporated into each matrix.

Nonetheless, matrices with *Aloe vera* gel showed an increase in the inhibition zone diameter, where A-AV and C-AV matrices improved their antibacterial capacity. This enhancement in antibacterial capacity of matrix A could be linked to the high amount of *Aloe vera* gel absorbed and delivered by the matrix, the high amount of silver nanoparticles present in the superior surface and its controlled release capacity of *Aloe vera* gel, during 400 min. On the other hand, the high antibacterial capacity of matrix C could be associated with a higher *Aloe vera* gel release capacity during the first minutes and the high ratio of chitosan (75%) present in the polymer mixture, increasing the ion content (NH_3_^+^) which are responsible for the antibacterial activity. 

The increase in antibacterial activity of *Aloe vera* gel loaded matrices could be associated to the presence of acemannan, anthraquinones and salicylic acid, which are responsible for its antibacterial properties [39,40]. Furthermore, matrices with *Aloe vera* gel could keep delivering gel for approximately 8 h, allowing the application of these compounds to the infected area with Gram (+) for a longer period of time.

**Figure 6:**
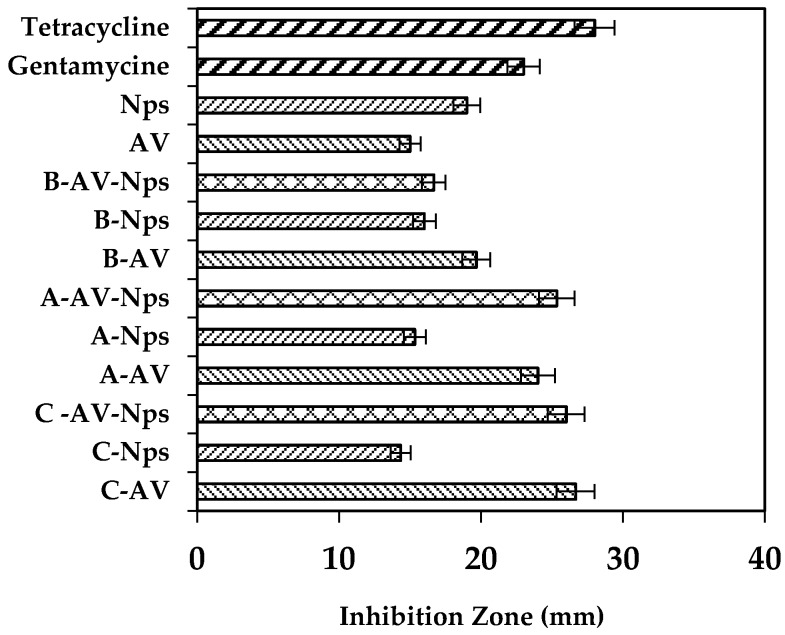
Inhibitory capacity against *S. aureus* of matrices control (A, B, C), matrices recharged with nanoparticles (A-Nps; B-Nps; C-Nps) and matrices recharged with *Aloe vera*/AgNps (A-AV-Nps; B-AV-Nps; C-AV-Nps).

As shown in Figure 6, when incorporating AgNps in the matrices (A-AgNps, B-AgNps and C-AgNps) they exhibited an inhibition zone, which indicates that there was antibacterial capacity against *S. aureus*. Comparing these matrices (A-AgNps, B-AgNps and C-AgNps) with matrices with *Aloe vera* (C-AV > A-AV > B-AV), findings show there were no significant differences among their inhibition zones (*t*-test, confidence level of 95%). This antibacterial capacity could be related to a synergy among *Aloe vera*, silver nanoparticles and the surface of the polymeric matrix.

Additionally, to verify the antibacterial capacity of each treatment, a comparison with two controls was performed: gentamicin and tetracycline. Treatments C-AV-Nps and A-AV-Nps showed values of the inhibition zone close to the value of the inhibition zone of tetracycline. Moreover, the B-AV-Nps treatments showed an inhibition capacity lower than tetracycline and the other two treatments (C-AV-Nps and A-AV-Nps).

Comparing the developed treatments with gentamicin, it was found that inhibition of B-AV-AgNps matrix was approximately close to the inhibition capacity of gentamicin, whereas C-AV-AgNps and A-AV-AgNps matrix showed an inhibitory capacity greater than gentamicin and closer to tetracycline.

The antibacterial property of matrices evaluated against *S. aureus* may be related to four important factors. The degree of acetylation of the chitosan, which was between 75% and 85%, provides a moderate antibacterial activity against *S. aureus* [41]; the high chitosan proportion in the polymer mixture, induces an increase in the groups (R-NH_3_^+^) in the matrix, promoting electrostatic interaction with negative charges on the cell wall of the bacteria affecting membrane permeability causing its death [41]. The swelling degree is related to the amount of *Aloe vera* gel absorbed at the time of charging and the silver nanoparticles having the ability to adhere to the cell wall, altering membrane permeability and cellular respiration [42,43].

**Figure 7:**
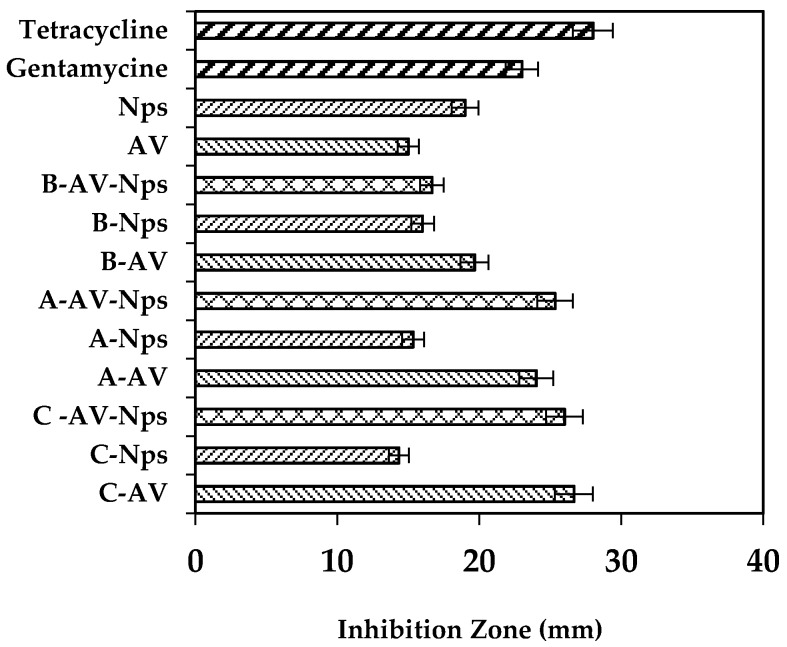
Inhibitory capacity against *P. aeruginosa* of matrices control (A, B, C), matrices recharged with nanoparticles (A-Nps; B-Nps; C-Nps) and matrices recharged with *Aloe vera*/AgNps (A-AV-Nps; B-AV-Nps; C-AV-Nps).

As in the previous case, control matrices were used without recharging, finding that A, B and C did not show antibacterial activity against *P. aeruginosa*. In addition, recharged matrices with only *Aloe vera* gel did not display an inhibition zone against it (data not shown). This behavior could be related to the low capacity of *Aloe vera* gel to inhibit *P. aeruginosa*. Nevertheless, when incorporating the AgNps into each treatment (B-AgNps, C-AgNps and A-AgNps) they showed an inhibition zone as shown in AgNps control solution. Figure 7 displays a similar dimension of the inhibition zone of the treatments (B-AgNps, C-AgNps and A-AgNps) as the matrix loaded with AgNps [39].

Figure 7 displays the inhibition zone of recharged matrices with AgNps and AV-AgNps. These results show that matrices with AgNps have the same property as gentamicin to inhibit Gram negative bacteria growth.

From the results above, it could be said that the antibacterial activity against *Pseudomonas* is due to the presence of the nanoparticles rather than to the incorporation of *Aloe vera* (*p* < 0.05, confidence level 95%). The implementation of the silver nanoparticles to each of the systems generates a bactericidal effect against Gram (-) and supplies Ag^+^ ions, which will interact with the membrane of the bacteria increasing their permeability and causing their death [39].

## 3. Materials and Methods 

### 3.1. Materials

Reactive grade sodium alginate from brown algae (low viscosity), chitosan from shrimp shells, ≥75% (deacetylated) (Sigma-Aldrich, San Luis, MO, USA), sodium bicarbonate, glacial acetic acid (Chemi, Productos Químicos Ltda, Bogotá, Colombia) and *Aloe vera* gel (obtained by filtration method) were used to obtain the polymeric systems. Silver Nanoparticles were supplied from Biomaterials Laboratory (Universidad EIA-CES, Envigado, Colombia), *Staphylococcus aureus* (ATCC 25923), *Pseudomonas aeruginosa* (ATCC 27853), tetracycline and gentamicine were used for antimicrobial testing.

### 3.2. Extraction of Aloe vera Gel

The extraction of the *Aloe vera barbadensis* gel, requires several leaves with a length between 40.0 cm and 50.0 cm. Subsequently, they were washed with distilled water, ethanol and hypochlorite to reduce contamination. The bark was then removed with a scalpel and the gel sheets were obtained, these were subject to a mechanical stirring process for 2 min to obtain a white juice, and then put through a filtration process. The first filtration was by gravity, obtaining a first solution, which was led to a vacuum filtration process, resulting in a gel with less solids that was used to recharge the polymer matrices.

### 3.3. Matrix Preparation

Chitosan powder (Chit) was dissolved in an acetic acid aqueous medium (0.2 M) to obtain a homogeneous solution at a concentration of 1.0% (*v*/*v*) (Chit 1) and 1.5% (*v*/*v*) (Chit 1.5). Likewise, alginate powder was dissolved in distilled water to obtain a homogeneous solution at a concentration of 1.0% (*v*/*v*) (Alg 1). Then, an alginate solution 1.0% (*v*/*v*) (Alg 1) was added to the chitosan solution 1.0% (*v*/*v*) (Chit 1) and 1.5% (*v*/*v*) (Chit 1.5) at a ratio of 1:3 (Chit 1/Alg 1), 1:3 (Chit 1.5/Alg 1) and 1:3 (Chit 1.5/Alg 1) as shown in Table 1, for a total of three systems: A (1:3 Chit 1/Alg 1), B (1:3 Chit 1.5/Alg 1) and C (3:1 Chit 1/Alg 1/B12) the last system contained NaHCO_3_ 0.12 M (B12) [36]. Later, each system was stirred at 4 °C for at least 2 h. After homogenization, the blended solutions were cast in Petri dishes, stored at 0 °C for 24 h and then lyophilized (L) for 24 h.

### 3.4. Loading of the Polymer Matrices with Aloe vera Gel and Incorporation of the Silver Nanoparticles

Matrices (dimensions of 1.0 cm × 1.0 cm) were immersed in 3 mL of *Aloe vera* gel for 24 h. Once the recharge time was completed, excess gel over the matrix surface was withdrawn. Each matrix was dipped in a solution of silver nanoparticles for 5 min, until a color change was observed in the sample. To determinate the amount of gel that was absorbed by the matrix, the percentage of swelling capacity was calculated using the Equation (1):

(1) $$\%\text{Swelling degree}=\frac{W_{1}-W_{0}}{W_{0}}\times 100\%$$

*W*_0_ = weight of dry matrix; *W*_1_ = weight of matrix after 24 h immersed in *Aloe vera* gel.

### 3.5. Matrix Characterization

#### 3.5.1. ATR Fourier Transform Infrared Spectroscopy (ATR-FTIR)

The infrared spectra of each sample was done in an FTIR spectrometer (Perkin-Elmer 1600 series). All spectra were obtained between 4000 cm^−1^ and 700 cm^−1^.

#### 3.5.2. SEM Analysis

The surface morphology of each matrix was determined by a Scanning Electron Microscopy JEOL JSM 6490 LVV (JEOL, Musashino, Akishima, Tokyo, Japan) using secondary electrons, EDS spectra were performed to determine the percentage of AgNps (silver nanoparticles) present on the surface of each matrix.

#### 3.5.3. Release Kinetics Assay

Three release kinetics assays were performed simultaneously by placing each of the samples into stainless steel dishes, then immersed in an 80 mL beaker with 40 mL of distilled water at pH 5.5, stirred constantly at 300 rpm and at a temperature of 37 °C. Subsequently, 10 mL aliquots were taken from each beaker every 5 min for the first 15 min, then every 15 min until completing one hour and from this time every half hour until completing five hours. This experiment was carried out under normal skin and body temperature conditions. During the experiment, the assays were carried out at 37 °C and pH 5.5 (48). The absorbance was measured on a UV-Visible Lambda 35 spectrophotometer (Perkin Elmer, Waltham, MA, USA) [1]. The results were analyzing with Peppas model showed in Equation (2):

(2) $$\frac{M_{t}}{M_{inf}}=Kt^{n},$$

where *M_t_* = fraction of drug released at time *t*, *M_inf_* = total amount of drug in the system, *K* = the release rate constant, *n* = release exponent.

#### 3.5.4. Antibacterial Activity

To determine the antibacterial ability of each treatment against *Staphylococcus aureus* (ATCC 25923) and *Pseudomona aeruginosa* (ATCC 27853,) Kirby Bauer assay was performed. Three samples of each system were subject to a recharging process with *Aloe vera* gel for 24 h, and subsequently 20 μL of silver nanoparticles were added. Five-asset controls were used: *Aloe vera* gel, nanoparticles solutions, dry matrices, gentamicin and tetracycline.

## 4. Conclusions

The alginate-chitosan matrices loaded with AgNps and *Aloe vera* gel, which were obtained with the technique proposed in this research, showed great capacity for absorbing *Aloe vera* gel, due to a porous structure with interconnected pores which facilitates the release of the gel. Each matrix exhibited different release kinetics depending on the alginate and chitosan content, particularly in matrix A, where the release of *Aloe vera* gel presented a Fickian behavior. The antibacterial capacity of the matrices loaded with *Aloe vera* and AgNps against *S. aureus* and *P. aueruginosa* was demonstrated, which could be used as an alternative for tetracycline and gentamicin.

The synergy between alginate, chitosan, *Aloe vera* gel and the AgNps offers a promising alternative to be used in antibacterial applications. This alternative method may help to decrease the secondary effects of antibiotics that are commonly used in wound treatments, with the advantage that the matrices developed promote wound healing through their chemical characteristics. This alternative must be studied and tested further in future research projects.

## Figures and Tables

**Table 1:** Abbreviation and composition of each matrix, their composition and proportion of alginate and chitosan.

Matrix	Alginate Solution 1% (*v*/*v*) (Alg1)	Chitosan Solution 1% (*v*/*v*) (Chit 1)	Chitosan Solution 1.5% (*v*/*v*) (Chit 1.5)	NaHCO_3_	*Aloe vera* Gel AV	Nanoparticles AgNps
A	75%	25%	*	*	*	*
B	75%	*	25%	*	*	*
C	25%	75%	*	0.12 M	*	*
A-AV	75%	25%	*	*	Yes	*
B-AV	75%	*	25%	*	Yes	*
C-AV	25%	75%	*	0.12 M	Yes	*
A-Nps	75%	25%	*	*	*	Yes
B-Nps	75%	*	25%	*	*	Yes
C-Nps	25%	75%	*	0.12 M	*	Yes
A-AV-Nps	75%	25%	*	*	Yes	Yes
B-AV-Nps	75%	*	25%	*	Yes	Yes
C-AV-Nps	25%	75%	*	0.12 M	Yes	Yes

* It does not contain.
